# Harmonious bilingual experience and child wellbeing: a conceptual framework

**DOI:** 10.3389/fpsyg.2023.1282863

**Published:** 2023-11-24

**Authors:** He Sun

**Affiliations:** National Institute of Education, Nanyang Technological University, Singapore, Singapore

**Keywords:** harmonious bilingualism, social-emotional wellbeing, child bilingualism, language output, shared book reading, dual language proficiency

## 1 Introduction

Compared to their monolingual peers, bilingual children may experience different developmental routes and rates in cognitive and social-emotional skills due to their unique dual language experience, which includes frequent language switching. Previous studies have predominantly focused on the relationship between child bilingualism and cognitive development, with relatively less attention given to the association between child dual language learning and social-emotional wellbeing (Halle et al., 2011). In fact, bilingual children must navigate between two sets of cultural expectations that have distinct goals for behavior that relates to social-emotional development (Halle et al., 2014). This negotiation of cultural expectations occurs for various reasons, across different aspects of life, and with a wide range of people (Grosjean, 2010).

Effective communication between bilingual children and key stakeholders (e.g., parents, peers, and teachers) equip children with better skills to adapt to new environments and reduces the risk of internalizing and externalizing issues (Han, 2010). Parents play a particularly important role during the process, as “children's socio-emotional skills are thought to originate in the home environment” (Farver et al., 2006, p. 198). Not only does parents' language use influence their children's language use, literacy practices, and language competence, it also passes on their cultural values and beliefs (Halle et al., 2014). As children's first teachers, their interaction with their children not only lays the basis for their learning of social norms and behaviors, but also helps children form their self-concept and develop social adjustment (Oller and Jarmulowicz, 2007). Therefore, it is important to investigate parents' language use and its antecedents, to explore their impact on children's bilingual experience. In this opinion paper, I propose a holistic framework on Harmonious Bilingual Experience (HBE), a concept derived from Harmonious Bilingual Development (De Houwer, 2015), to address:

1) How parents' bilingual perception and proficiency may influence their language use,
2) How parental language use would influence their children's language use, literacy activities, and dual language proficiency,
3) How children's bilingual experience is related to their language and social-emotional skills.

As the relationship between children's HBE and their social-emotional wellbeing is the focus of our discussion, we review the major findings about this relationship first, before discussing parental factors. To conclude, I present a four-tiered conceptual framework to link the three parts of the review, addressing the relationship between parental language perception and proficiency, parent and child language use, and child social emotional wellbeing.

## 2 Child bilingual experience and their social-emotional wellbeing

Social-emotional wellbeing is a comprehensive concept that encompasses interrelated areas of social and emotional competence, including skills related to social interactions such as self-regulation, conflict resolution, and the establishment and maintenance of positive peer relationships (SAGE Reference, 2016). The literature reveals that both internal and external factors can affect the social-emotional and behavioral skills of bilingual children (Han, 2010; Sun et al., 2021). In this paper, I will specifically focus on three components: language use, exposure to literacy, and bilingual proficiency.

### 2.1 Child dual language use

Vygotsky (1962) conceptualized language as a vital social tool learned and developed through interactions with others. Children's use of language in interactions with various interlocutors is the primary means for them to grow in both linguistic and social competence. Bilingual children navigate a complex social world, where individuals in their social network possess differing language knowledge (Byers-Heinlein and Lew-Williams, 2013). Furthermore, they are likely to be exposed to a variety of socialization practices through language from parents and caregivers. In comparison to their monolingual peers, bilingual children may excel in differentiating between these socialization cues and responding appropriately in diverse social contexts (Halle et al., 2014). This, in turn, facilitates communication and fosters better relationships with peers, teachers, and family (Han, 2010). The more opportunities bilingual children have for communication, the greater their chances of developing social skills (Coelho et al., 2018), forming a positive feedback loop (Gallagher, 1999).

Empirical studies have demonstrated this positive link. In a study of 805 Singaporean bilingual preschoolers, Sun et al. (2021) discovered that the number of months children had been speaking both of their languages was significantly and positively related to their prosocial skills, even after controlling for multiple covariates such as socioeconomic status and gender. Similar evidence has been found for immigrant children, whose use of the societal dominant language is associated with positive relationships with peers (Chen and Tse, 2010) and teachers (Ren and Wyver, 2016). Beyond the use of societal dominant languages, children's heritage languages are critical for their social-emotional development. Tannenbaum and Howie (2002) found that children's use of heritage languages at home was associated with their perception of their family as cohesive and egalitarian. Children who perceived their families in this way were more likely to speak and maintain their parents' language.

### 2.2 Home literacy activities

Home literacy activities, book reading in particular, have been shown to be connected to child social-emotional skills as well. The positive association is supported by Farver et al.'s (2006) finding, who found that low SES Latino mothers' literacy involvement (e.g., home reading frequency), was linked to their children's better social competency in the US. Sun (2019) confirmed the results, finding that Chinese literacy activities such as library visits and parent-child shared reading sessions were associated with lower child difficulty level and better prosocial skills in Singapore.

The benefits of reading for social-emotional wellbeing may stem from the content of children's books, as well as the nature of the activity. Books targeted at children are often rich in socio-emotional content: Dyer et al. (2000) found that in 90 children's books, on average, a reference to social events or emotions occurred every three sentences. These books are often centered around interactions between people or personifications and are a good source of exposure to emotional states and social situations (Aram and Aviram, 2009). Looking at illustrations in these books also gives young readers the opportunity to reflect on and discuss the behaviors, feelings, relationships, and differing intentions and perspectives of book characters (Murray et al., 2016; Sun, 2022). Additionally, adults often discuss important social-emotional concepts (e.g., sharing) with children during shared book reading (Sun, 2022), which can help children better understand these notions. Home literacy activities are thus important bases where children are exposed to social norms and moral behavior and develop the language skills that they can use to enact said norms and behaviors. While it is crucial, it should be noted that a literary tradition in the heritage language might not be readily available across all ethnicities. Therefore, bilingual parents may not be able or willing to read in the majority language in some cultures.

### 2.3 Dual language proficiency

Language ability is positively associated with children's social competence: As children grow in language proficiency, so does their ability to use the language to communicate. For bilingual children, the ability to communicate in both societal and home languages is important to their social emotional wellbeing (Han, 2010; Sun et al., 2021). At home, the use of heritage languages is believed to be conducive to good family relationships (Ren and Wyver, 2016). Boutakidis et al. (2011) found that adolescents' heritage language fluency among Chinese and Korean immigrant families was positively related to their respect for parents, a relationship mediated by quality of communication. The authors argue that these positive effects go beyond simple communication. Language is the main tool that parents can use to convey cultural values and beliefs (Halle et al., 2014), including terms and concepts unique to heritage languages such as honorifics or titles of respect (Boutakidis et al., 2011). Sharing a language can improve the quality of communication, promoting cultural understanding and a shared view of the world.

In the school setting, children's societal language proficiency seems to be important for obtaining peer acceptance and getting involved in peer activities (Chen and Tse, 2010). Pallotti (1996) presents the example of a Moroccan girl in an Italian preschool, whose early Italian language proficiency consisted largely of phrases that would gain her access to peer interaction. Children who do not speak the societal language well often face bullying and victimization by their monolingual peers (De Houwer, 2020), which can cause psychological harm. For instance, Hispanic bilingual children with lower English proficiency in kindergarten were found to exhibit more externalizing behaviors than their peers with higher English proficiency (Dawson and Williams, 2008).

## 3 Child bilingual experience and their parental language use, perception, and proficiency

The three key bilingual factors (i.e., child dual language use, literacy practice, and bilingual proficiency) are positively associated with children's home and school language environment (De Houwer, 2018; Sun et al., 2020; Luo and Song, 2022), as well as child agency (Schwartz and Mazareeb, 2023). Among which, parents play a critical role in children's early bilingual development. Various studies reveal that parents' and children's current language use patterns are highly correlated (e.g., Bedore et al., 2012; Sun et al., 2021, 2022), and parental language use is also significantly associated with the home literacy environment: those who speak to their children more in a language also tend to conduct more literacy activities in that language (e.g., Baker, 2014). Lastly, the quantity and quality of parental language use has also been consistently found to affect bilingual children's semantic and morphosyntactic development (Cobo-Lewis et al., 2002).

Various factors, including familial socio-economic status, can influence parents' language input (Hoff, 2005). Among which, parental language perception and proficiency have been consistently found to influence the quantity and quality of parental language use (De Houwer, 1999; Paradis, 2011; Surrain, 2021). De Houwer (1999) emphasizes the importance of parental attitudes and impact belief, which affects their language choices and interaction strategies used with their children, which in turn affects their children's language development. Parents who harbor positive attitudes toward bilingualism are believed to aid their children's bilingual development (De Houwer, 1999). Although being crucial, positive bilingual perception alone is insufficient to promote HBE. Curdt-Christiansen (2016) presented a case where a parent mainly spoke English to their children at home, despite claiming positive attitudes toward Malay language.

One possible reason for this could be parental language proficiency. While parents may want to speak to their children in a certain language, they may lack the proficiency to do so regularly. Sun et al. (2022) found that amongst English–Mandarin bilingual mothers in Singapore, a high self-evaluated Mandarin proficiency corresponded to a significantly higher amount of Mandarin spoken to their children compared to low or medium proficiency mothers. Low and medium proficiency mothers tended to use significantly more English than Mandarin, compared to high proficiency mothers.

Parental proficiency could also affect the quality of their input. Mothers with high L2 proficiency were found to have a larger vocabulary size (Bialystok, 2009) and use more varied and complex words with their children (Rowe, 2012; Hoff et al., 2020). Their proficiency has been found to affect children's literacy activity. Baker (2014) found those mothers who were more proficient in English engaged in more English literacy activities with their children such as singing songs, shared book reading, or visiting the library.

## 4 Harmonious bilingual experience: a four-tiered conceptual framework

**Figure 1:**
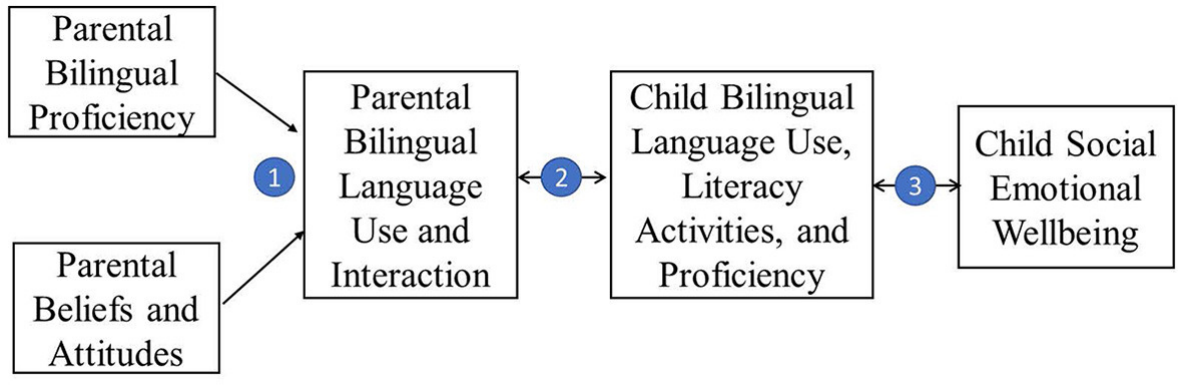
Harmonious bilingual experience: a four-tiered conceptual framework.

The review above leads to a four-tier conceptual framework demonstrated in Figure 1. In the bilingual home setting, parent's perception of bilingualism and their proficiency in dual languages impact the extent of their language use with their children, which in turn influences their children's bilingual use, literacy activities, and dual language skills. These three aspects of children's bilingual experience eventually impact their social-emotional wellbeing, such as prosocial skills and emotion recognition, even controlling the direct impact of parental social-emotional wellbeing. The current four-tier conceptual framework calls for large-scale and longitudinal studies to verify the potential chain of effects unidirectionally or bidirectionally. It highlights the necessity of doing research on early bilingualism across domains, with united efforts from linguists, developmental psychologists, and educators. It is worth noting that other distal (e.g., educational policy, familial SES) and proximal factors (e.g., child's communicative needs) would also affect the chain of effect and future research should control these covariates when examining the framework.

## Author contributions

HS: Conceptualization, Validation, Writing – original draft, Writing – review & editing.
